# Web-Based Guide to Health: Relationship of Theoretical Variables to Change in Physical Activity, Nutrition and Weight at 16-Months

**DOI:** 10.2196/jmir.1614

**Published:** 2011-03-04

**Authors:** Eileen Smith Anderson-Bill, Richard A Winett, Janet R Wojcik, Sheila G Winett

**Affiliations:** ^3^PC Resources IncBlacksburg, VAUnited States; ^2^Exercise Science ProgramDepartment of Physical Education, Sport & Human PerformanceWinthrop UniversityRock Hill, SCUnited States; ^1^Center for Research in Health BehaviorDepartment of PsychologyVirginia TechBlackburg, VAUnited States

**Keywords:** Internet users, dietary habits, physical activity, psychosocial aspects, self-efficacy, social support, self-regulation

## Abstract

**Background:**

Evaluation of online health interventions should investigate the function of theoretical mechanisms of behavior change in this new milieu.

**Objectives:**

To expand our understanding of how Web-based interventions influence behavior, we examined how changes at 6 months in participants’ psychosocial characteristics contributed to improvements at 16 months in nutrition, physical activity (PA), and weight management as a result of the online, social cognitive theory (SCT)-based Guide to Health intervention (WB-GTH).

**Methods:**

We conducted recruitment, enrollment, and assessments online with 272 of 655 (41.5%) participants enrolling in WB-GTH who also completed 6- and 16-month follow-up assessments. Participants’ mean age was 43.68 years, 86% were female, 92% were white, mean education was 17.45 years, median income was US $85,000, 84% were overweight or obese, and 73% were inactive. Participants received one of two equally effective versions of WB-GTH. Structural equation analysis of theoretical models evaluated whether psychosocial constructs targeted by WB-GTH contributed to observed health behavior changes.

**Results:**

The longitudinal model provided good fit to the data (root mean square error of approximation <.05). Participants’ weight loss at 16 months was predicted by improvements in their PA (beta_total_ = -.34, *P* = .01), consumption of fruits and vegetables (F&V) (beta_total_ = -.20, *P* = .03) and calorie intake (beta_total_ = .15, *P* = .04). Improvements at 6 months in PA self-efficacy (beta_total_ = -.10, *P* = .03), PA self-regulation (beta_total_ = -.15, *P* = .01), nutrition social support (beta_total_ = -.08, *P* = .03), and nutrition outcome expectations (beta_total_ = .08, *P* = .03) also contributed to weight loss. WB-GTH users with increased social support (beta_total_ = .26, *P* = .04), self-efficacy (beta_total_ = .30, *P* = .01), and self-regulation (beta_total_ = .45, *P* = .004) also exhibited improved PA levels. Decreased fat and sugar consumption followed improved social support (beta_total_ = -.10, *P* = .02), outcome expectations (beta_total_ = .15, *P* = .007), and self-regulation (beta_total_ = -.14, *P* = .008). Decreased calorie intake followed increased social support (beta_total_ = -.30, *P* < .001). Increased F&V intake followed improved self-efficacy (beta_total_ = .20, *P* = .01), outcome expectations (beta_total_ = -.29, *P* = .002), and self-regulation (beta_total_ = .27, *P* = .009). Theorized indirect effects within SCT variables were also supported.

**Conclusions:**

The WB-GTH influenced behavior and weight loss in a manner largely consistent with SCT. Improving social support, self-efficacy, outcome expectations, and self-regulation, in varying combinations, led to healthier diet and exercise habits and concomitant weight loss. High initial levels of self-efficacy may be characteristic of Web-health users interested in online interventions and may alter the function of SCT in these programs. Researchers may find that, although increased self-efficacy enhances program outcomes, participants whose self-efficacy is tempered by online interventions may still benefit.

**Trial Registration:**

Clinicaltrials.gov NCT00128570; http://clinicaltrials.gov/ct2/show/NCT00128570 (Archived by WebCite at http://www.webcitation.org/5vgcygBII)

## Introduction

As many as 79% of Americans use the Internet, with growing majorities across racial, ethnic, educational, and income groups reporting going online – 73% go online daily. The vast majority of users go to the Internet for information on health topics [1,2]. Internet-based health information has largely been viewed as positive, influencing health decisions and changing the way users think about diet and exercise [3]. With almost universal Internet access and adoption, reach, effectiveness [4], and the function of theoretical mechanisms [5-9] become pivotal issues facing Internet-based public health interventions. 

Randomized control trials of Internet-based interventions have largely produced modest, short-term effects [10-17]. Others have shown sustained effects on nutrition and physical activity (PA) for longer periods (ie, 16 months) [18] with results comparable in many ways to results from more intensive, face-to-face interventions targeting lifestyle behaviors [19,20]. None of these trials, however, has recruited participants, delivered programs, and evaluated outcomes entirely online, as is needed to establish the effectiveness of Web-contained interventions.

In addition to limited evidence of long-term effectiveness, attrition is typically high in Internet-based trials (43%-50%) [4], with tentative users who attempt but quickly withdraw from the intervention, short-term users who seem to drop out after using the program for a short time, and stable users who stick with a program over the long term [21]. A presumed advantage of the Internet, however, is the ability to provide a high-fidelity intervention at virtually any dose level, allowing participants to tailor program use to their specific needs and circumstances [4]. Although some suggest that the effectiveness of programs is best reflected in the effects on stable users [21], including short-term users in evaluations provides a more accurate reflection of overall program impacts [22].

The reach of entirely online interventions is defined as the percentage and representativeness of individuals willing to participate [23] from the number of people who arrive at a site either through a search engine or by directly entering the website address. Website use, in combination with username/password entry, allows researchers to capture the number exposed to each component of recruitment, enrollment, and, ultimately, of the intervention [4].

**Figure 1:**
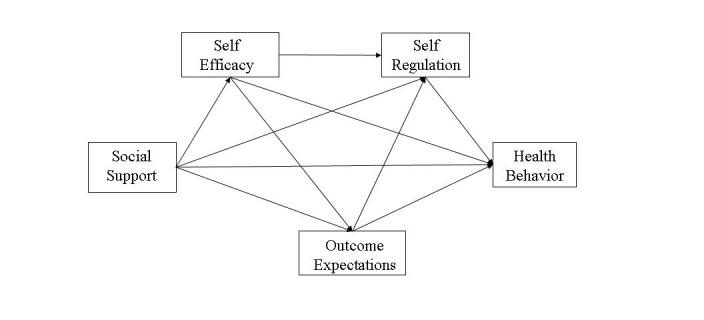
Social cognitive theory of health behavior

Finally, Internet-based health promotion interventions should be based on theory and evaluated in a way to validate and refine the application of theory within the new delivery environment [5-7,24,25]. Social cognitive theory (SCT; Figure 1) [26,27] suggests that Internet health interventions must help individuals develop a sense of self-efficacy in specific behaviors (such as being physically active and eating nutritiously), which promotes individuals’ positive expectations for behavior change and their modification or differential use of self-regulatory skills (ie, planning, self-monitoring, problem solving, and setting self-standards, goals, and self-incentives). SCT further suggests that, as a precursor to self-efficacy, social support enhances the process of behavior change and maintenance. 

The current study examined the relationship of SCT variables to behavior and weight change in the Web-Based Guide to Health intervention (WB-GTH). An earlier site-based version of GTH suggested that self-efficacy, self-regulation, and social support (and, to a lesser extent, outcome expectations) can mediate the effects of SCT-based health interventions in a manner consistent with theory [28]. It is not clear, however, how such interventions, implemented over a longer period (52 weeks vs 12 weeks) and entirely online with no face-to-face contact with researchers, no on-site computer support, and no direct manipulation of essential environmental support (social and physical), might influence behavior.

The WB-GTH featured online recruitment, screening, consent, assessment, and program implementation with and without enhanced self-regulatory components (basic WB-GTH and enhanced WB-GTH versions). Recruitment into the WB-GTH, described in detail elsewhere [29], included Web-browser advertisements and direct mailings, but was successful largely through newsletters (print and electronic) and listservs of existing organizations. Most participants attracted to the WB-GTH website registered for eligibility screening (85%, [29]). Retention rates from being eligible to providing informed consent and from providing consent to completing baseline were 74%-75% [29].Similar to other efforts [30,31], WB-GTH attracted mostly middle-aged, well-educated, upper-middle-class women. Following study requirements, the sample population was inactive to sedentary and mildly overweight to obese, but otherwise healthy. Psychosocial characteristics of WB-GTH enrollees suggested social support and self-efficacy for behavior change and enhanced self-regulatory skills would be important to developing healthier levels of nutrition and PA [29]. Both basic WB-GTH and enhanced WB-GTH resulted in significant improvements in nutrition, PA, and weight management [32].

The purpose of the current study was to determine whether improvements at 6 months in social support, self-efficacy, outcome expectations, and self-regulation preceded observed behavior and weight change in a manner consistent with SCT [26].

## Methods

### Recruitment and Participants

The WB-GTH trial was conducted entirely online (baseline assessment from September 2007 to November 2008; 6-month assessment from April 2008 to May 2009, 16-month assessment from February 2009 to March 2010). Of 655 randomly assigned participants, 199 quickly withdrew from the program, logging in to only one or two program modules. Participants who quickly withdrew from WB-GTH were less active, but otherwise did not differ in demographics or behavior from those who engaged with one of the two versions of the WB-GTH (ie, saw at least three program modules; n = 456; [32]). Of these engaged participants, 59.6% returned for the 16-month assessment and were included in the current study (272/655 total randomized participants, 41.5%). Included participants had a mean age of 43.68 (SD 10.39) years, 86% were female, and 92% were white. The sample was well educated with a mean of 17.45 (SD 3.0) years completed, and had a median annual household income of about US $85,000; 84% were overweight or obese and 73% were inactive (ie, <7500 steps/day; mean 6178.15, SD 1825.39). The 184 engaged participants who did not return for follow-up assessment did not differ on demographic or baseline outcome variables from participants included in the current study except that they had slightly higher body mass indexes than included participants (mean 30.36, SD 4.22 vs mean 29.18, SD 3.83).

### The WB-GTH Intervention

The WB-GTH program (described in detail elsewhere [32]) ran for 52 weeks with SCT-based modules each consisting of 15-20 Web screens requiring participants to be online for 5-10 minutes each week. Participants logged in as often as once a week with the username/password they created during enrollment. Early modules targeted self-efficacy through gradual behavior change guided by self-regulation. WB-GTH next presented a series of core-content modules [32] that additionally addressed social support and outcome expectations related to behavior change. After 4 months, the WB-GTH focus shifted to behavior-change maintenance with continued self-regulation.

The basic WB-GTH program, used by 51.1% (139/272) of current study participants, provided generic goals for adding steps and minutes of walking to their daily routines (ie, add 400 steps/day each week up to 3000 steps and 5 minutes/day up to 30 minutes, 5 days/week). Other goals included adding fitness walking after reaching 30 minutes of walking 5 days a week, adding fruits and vegetables (F&V) (1/day each week to reach 5-9 for female and 5-10 for male users), adding whole-grain foods and low-fat dairy foods (1/day each week for up to 3 servings a day), and decreasing high-fat and high-sugar foods (no more than 28 servings/week). Participants kept and reported daily logs of steps, minutes walked, enjoyment of and exertion during planned walks, and intake of fruits, vegetables, whole grains, low-fat dairy, and high-fat and high-sugar foods, and they weighed themselves weekly. Each week users of the basic WB-GTH program received general feedback (eg, comparison of current levels and overall goals) with a restatement of generic goals and a prompt for users to plan for the next week. The enhanced WB-GTH program, used by 48.9% (133/272), differed from the basic program by providing users with personalized feedback and tailored goal setting and planning, and by allowing participants to select, report, and receive feedback on specific behavior-change strategies for increasing PA and improving nutrition [32].

### Measures

#### Nutrition

Participants completed the Block 2005 Food Frequency Questionnaire (NutritionQuest, Berkeley, CA, USA) [33] online. We examined the resulting estimates of daily intake in kilocalories, percentage kilocalories from fat and from sugar, and daily servings of fruits and of vegetables at baseline and 16 months and change during the 16-month interval (ie, 16-month assessment minus baseline).

#### PA and Body Weight

Participants used a pedometer (Digi-walker SW-200, Yamax USA, Inc, San Antonio, TX, USA) and a digital bathroom scale (Health-o-meter HDL150-01, Sunbeam Products, Inc, Maitland, FL, or Tanita HD-313, Tanita Corporation, Arlington Heights, IL, USA) provided at baseline. Participants wore their pedometers for 7 days and logged the number of steps registered each day. They returned to the WB-GTH website to report daily steps for at least 4 consecutive days. Participants also completed an online questionnaire about the duration, pace, and number of walking, treadmill, jogging, and running sessions they took in a typical week. The metabolic equivalent (MET; ie, the ratio of work metabolic rate to a standard resting metabolic) for each activity was computed and summed across activities to compute walking METhours/week for each participant at baseline and 16 months. Participants used the bathroom scale provided to measure body weight in pounds without clothing just after waking in the morning or before going to bed in the evening. They reported their weight and height online along with their walking log data. We examined mean daily steps (total steps reported divided by days of pedometer use), mean walking METhours/week, body weight in pounds, and change in these variables during the 16-month interval. 

#### Social Cognitive Variables

The Health Beliefs Survey administered online at baseline and 6 months measured change in nutrition- and PA-related social support, self-efficacy, outcome expectations, and self-regulation (see Table 1).

### Statistical Analysis

Multivariate repeated measures analysis of variance (MANOVA) evaluated effects of the WB-GTH on SCT variables at 6 months, and nutrition, PA, and body-weight variables at 16 months. Full information maximum likelihood (FIML) latent-variable structural equation modeling (LISREL version 8.81, Scientific Software International, Inc, Lincolnwood, IL, USA) [34] evaluated the relationships among SCT-change variables at 6 months and behavioral and weight-change variables at 16 months. SCT fit was evaluated with root mean square error of the approximation (RSMEA) ≤.05 (*P*-value close fit > .95 or alpha = .05) and FIML chi-square was evaluated with alpha set at .05 or less than 3 times the degrees of freedom (normed chi-square) [35]. With few exceptions, the distributions of measure scores were skewed or displayed unacceptable kurtosis; we normalized measures using the Blom proportional estimate formula in SPSS version 17.0 (IBM Corporation, Somers, NY, USA). Additional variables were similarly normalized to retain a consistent unit of measurement within latent variables. Error variances for single-indicator latent variables (ie, weight change, PA self-efficacy, nutrition negative outcome expectations, and change in daily calorie consumption) were set to sigma^2^ × (1 - reliability). Error variances of PA- and nutrition-related SCT variables (eg, PA self-efficacy and nutrition self-efficacy) were allowed to correlate [35]. Error covariances between measured nutrition variables and between theoretically consistent SCT variables were allowed to correlate to improve model fit.

**Table 1:** Health Beliefs Survey: scale descriptions and internal consistency estimates

Variable description		Subscale	Number of items	Alpha^a^
**Food Beliefs Survey**				
	Social support	Family	11	.90
		Friends	11	.94
	Self-efficacy	Eating healthy foods	12	.91
		Avoiding high-fat and high-sugar foods	15	.90
		Planning and tracking intake	10	.96
	Positive physical and self-evaluative outcome expectations		10	.90
	Negative physical, social and self-evaluative outcome expectations		11	.89
	Self-regulation	Planning and tracking	11	.92
		High-fat and high-sugar foods	13	.90
		Healthy food choices	8	.90
**PhysicalActivity Beliefs Survey**				
	Social support	Family	8	.94
		Friends	8	.96
	Self-efficacy		23	.95
	Positive outcome expectations	Physical	5	.83
		Affective	5	.66
	Negative outcome expectations: physical, social, and self-evaluative		7	.88
	Self-regulation	Setting goals and planning activity	9	.91
		Tracking physical activity	5	.85
		Increasing enjoyment	3	.77

^a^ Cronbach alpha coefficient of internal consistency.

## Results

### WB-GTH Outcomes

Participants viewed a mean of 21.33 (SD 17.19) modules: 18% (49/272) viewed only introductory WB-GTH modules (ie, modules 1-5), 37.1% (101/272) also viewed WB-GTH core-content modules (6-16), and the remaining 44.8% (122/272) viewed maintenance modules (>17 modules). Participants viewed similar numbers of modules of each version: basic WB-GTH (mean 22.36, SD 17.51) and the enhanced WB-GTH (mean 20.34, SD 16.87; F_1,270_ = 0.95, *P* = .329).

MANOVA of baseline and 16-month data indicated that WB-GTH participants viewing both versions of WB-GTH made behavioral and weight changes (time: F_8,157_ = 10.02, partial-eta squared = .34, *P* < .001; version × time: F_8,167_ = 1.57, partial-eta squared = .07, *P* = .14). WB-GTH users increased daily steps, METhours/week expended in walking, and intake of fruits and intake of vegetables. WB-GTH users also decreased their intake of fat, sugar-sweetened foods, and calories (see Table 2). Improvements in nutrition and PA did not vary across the number of program modules viewed by participants (time × modules: F_1__6,316_ = 1.00 partial-eta squared = .05, *P* = .45).

Participants’ improvements in behavior and weight at 16 months were preceded by changes at 6 months in social cognitive characteristics related to nutrition (time: F_11,235_ = 42.91, partial-eta squared = .67, *P* < .001; version × time: F_11,235_ = 1.278, partial-eta squared = .06, *P* = .24) and PA (time: F_9,237_ = 90.15, partial-eta squared = .77, *P* < .001; version × time: F_9,237_ = 0.84, partial-eta squared = .03, *P* = .58). Enhanced social cognitive characteristics (see Table 2) included increased dietary social support from family members and friends, self-efficacy for eating healthier foods, and, albeit marginally, self-efficacy for reducing fat and sugar intake; decreased negative outcome expectations; and increased use of self-regulatory strategies related to eating F&V and whole grains, reducing fat and calories, and planning and tracking nutrition. Positive outcome expectations related to nutrition behavior did not change. WB-GTH users also improved perceived PA social support from family and friends and increased their use of self-regulation strategies. Self-efficacy for overcoming barriers to PA, however, significantly decreased as participants used the WB-GTH program. Outcome expectations related to PA did not change.

**Table 2:** Baseline, 6-month, and 16-month follow-up weight, physical activity, and nutrition-related outcomes of users of the Web-Based Guide to Health intervention

Outcome		Study time point^a^	Mean	SD	F	df^b^	*P*-value	Partial-eta squared^c^
**Body weight inpounds**		base	176.98	28.03	29.01	1,194	<.001	.130
		16 mo	172.34	28.01				
**Nutrition**								
	Fruit servings/day	base	1.13	0.81	83.34	1,236	<.001	.261
		16 mo	1.66	0.95				
	Vegetable servings/day	base	2.97	1.93	55.128	1,236	<.001	.189
		16 mo	3.95	2.36				
	Percentage kilocalories from fat	base	36.62	5.86	37.33	1,236	0.00	.14
		16 mo	34.40	6.21				
	Percentage kilocalories from sweets	base	14.53	9.32	45.07	1,236	0.00	.16
		16 mo	11.30	8.50				
	Kilocalories/day	base	1820.95	700.81	22.36	1,236	0.00	.09
		16 mo	1641.14	510.47				
	Family social support	base	2.70	.81	57.193	1,245	<.001	.189
		6 mo	3.07	.83				
	Friend social support	base	2.91	.78	29.768	1,245	<.001	.108
		6 mo	3.16	.74				
	Self-efficacy for avoiding fat and sugar	base	75.99	17.29	2.875	1,245	.091	.012
		6 mo	77.59	16.81				
	Self-efficacy for eating healthy foods	base	73.48	16.80	3.794	1,245	.053	.015
		6 mo	75.59	17.73				
	Positive outcome expectations	base	4.32	.61	0.01	1,245	0.95	.00
		6 mo	4.32	.57				
	Negative outcome expectations	base	2.90	.73	16.89	1,245	0.00	.06
		6 mo	2.72	.81				
	Self-regulation healthy food choices	base	3.11	.82	301.23	1,245	0.00	.55
		6 mo	4.06	.62				
	Self-regulation high-fat/high-sugar foods	base	3.01	.75	292.27	1,245	0.00	.54
		6 mo	3.84	.61				
	Self-regulation planning and tracking nutrition	base	2.35	.81	376.72	1,245	0.00	.61
		6 mo	3.58	.79				

**Physical activity**								
	Steps/day	base	6252.89	1876.34	40.32	1,177	0.00	.19
		16 mo	7741.47	3247.56				
	METhours/week walking^d^	base	2.82	9.70	45.20	1,177	0.00	.20
		16 mo	12.43	18.02				
	Family social support	base	2.49	1.03	19.98	1,245	0.00	.08
		6 mo	2.77	1.01				
	Friend social support	base	2.88	.98	8.05	1,245	0.01	.03
		6 mo	3.07	.87				
	Self-efficacy	base	64.09	19.24	4.06	1,245	0.05	.02
		6 mo	61.28	22.24				
	Positive affective outcome expectations	base	16.24	3.92	0.23	1,245	0.63	.00
		6 mo	16.38	4.57				
	Positive physical outcome expectations	base	20.49	4.22	2.33	1,245	0.13	.01
		6 mo	20.05	4.81				
	Negative outcome expectations	base	10.61	4.84	0.01	1,245	0.93	.00
		6 mo	10.65	5.13				
	Self-regulation goal setting and planning	base	1.98	.76	439.28	1,245	0.00	.64
		6 mo	3.47	.94				
	Self-regulation tracking activity	base	1.53	.71	604.49	1,245	0.00	.71
		6 mo	3.65	1.19				
	Self-regulation increase enjoyment	base	1.79	.84	387.59	1,245	0.00	.61
		6 mo	3.30	1.04				

^a^ Baseline (base), 6 months (6 mo), or 16 months (16 mo).

^b^ df: degrees of freedom for F test result.

^c^ partial-eta squared.

^d^ MET: metabolic equivalent.

### Social Cognitive Model of Change

**Figure 2:**
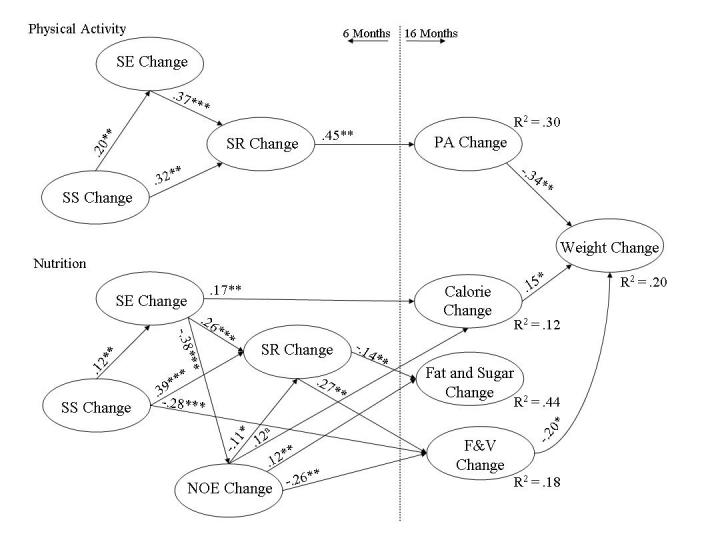
Social cognitive model of behavior and weight change among users of the Web-based Guide to Health intervention. F&V: fruits and vegetables; NOE: negative outcome expectations; PA: physical activity; SE: self-efficacy; SR: self-regulation; SS: social support. ^a^ *P* < .10; *P < .05; **P < 0.1; ***P < .001

A longitudinal, latent variable, structural model incorporated data from SCT variables exhibiting significant change (see Table 2) at 6 months, and nutrition, PA, and weight change at 16 months (Figure 2). The model provided good fit to the WB-GTH change data: root mean square error of approximation (RMSEA) = .05, 95% confidence interval 0.04-0.06; *P* (close fit: RMSEA < .05) = .33; FIML chi-square df = 200, n = 272) = 342.7, *P* < .001; chi-square/degrees of freedom ratio = 1.71). Means, standard deviations, and covariances of measured variables are available from Dr Eileen Anderson-Bill on request. 

#### Physical Activity

Completely standardized significant direct effect coefficients resulting from the structural analysis appear next to their associated paths; the PA portion of the model is at the top of Figure 2 (note: as reported in Table 2, outcome expectations did not change with use of the WB-GTH, and thus were not modeled as predicting change in PA). The associated R^2^ values indicated the model explained 30% of the variance of change in PA. Improvements at 6 months in social support, self-efficacy, and self-regulation led to increases in PA at 16 months (social support beta_total_ = .26, *P* = .04 self-efficacy beta_total_ = .30, *P* = .01; self-regulation: beta_total_ = .45, *P* = .004). Within the SCT variables, the indirect effects of self-efficacy (beta_indirect_ = .17, *P* = .007) and social support (beta_indirect_ = .20, *P* = .004) on PA provide evidence for the posited meditational roles of self-efficacy and self-regulation. Increased social support at 6 months led to higher PA at 16 months largely by increasing participants’ self-efficacy (beta_total_ = .20, *P* =.002) and self-regulation (beta_total_ = .39, *P* < .001). Similarly, participants’ increased self-efficacy contributed to higher levels of PA by making it more likely that participants would engage in self-regulatory behaviors (beta_total_ = .37, *P* < .001).

#### Nutrition

The nutrition portion of the model is at the bottom of Figure 2. The R^2^ values indicated the model explained 18% of the variance of change in F&V, 12% of change in calories, and 44% of change in fat and sugar-sweetened foods. Decreased fat and sugar consumption was preceded by improvements at 6 months in participants’ social support (beta_total_ = -.10, *P* =.02), negative outcome expectations (beta_total_ = .15, *P* = .007), self-regulation (beta_total_ = -.14, *P* = .008), and, albeit marginally, self-efficacy (beta_total_ = -.06, *P* = .07). Decreased calorie intake at 16 months was preceded by increased social support at 6 months (beta_total_ = -.30, *P* < .001). Increased F&V intake was associated with improved self-efficacy *(*beta_total_ = .20, *P* = .01), negative outcome expectations (beta_total_ = -.29, *P* = .002), and self-regulation (beta_total_ = .27, *P* = .009). The indirect effects of social support and self-efficacy on increased F&V (social support: beta_indirect_ = .14, *P* =.005; self-efficacy: beta_indirect_ = .18, *P* < .001) and decreased fat and sugar-sweetened foods (social support: beta_indirect_ = -.07, *P* =.01; self-efficacy: beta_indirect_ = -.09, *P* =.005) support meditational roles for negative outcome expectations and self-regulation in the nutrition portion of the model. 

#### Weight Management

The model also evaluated whether participants’ weight loss at 16 months related to concomitant changes in nutrition and PA or to psychosocial changes that preceded dietary and PA improvements. The model explained 20% of the variance of change in weight. Participants’ weight loss at 16 months was related to increases in PA (beta_total_ = -.34, *P* = .01), which mediated the effects on weight loss of improvements in PA self-efficacy (beta_total/indirect_ = -.10, *P* = .03) and PA self-regulation (beta_total/indirect_ = -.15, *P* = .01). PA self-efficacy also influenced weight loss through its effect on self-regulation (beta_total/indirect_ = .37, *P* < .001). Further, weight loss at 16 months was related to improved F&V intake (beta_total_ = -.20, = .04) and calorie consumption (beta_total_ = .15, *P* = .04), which mediated the effects of changes in nutrition-related social support (beta_total/indirect_ = -.08, *P* = .03), negative outcome expectations (beta_total/indirect_ = .08, *P* = .03), and, albeit marginally, self-regulation (beta_total/indirect_ = -.06, *P* = .07). Users of the WB-GTH lost weight because of improved diet and PA, which followed improvements in participants’ psychosocial characteristics. 

## Discussion

The WB-GTH is an Internet intervention designed to improve nutrition and PA and prevent further weight gain in overweight to obese, inactive, but otherwise healthy adults. Based on SCT, the WB-GTH was delivered with and without enhanced self-regulatory features to Internet users who were recruited, screened, asked for consent, and assessed entirely online. The 16-month outcomes suggest that WB-GTH users lost almost 5 pounds (~3%) of body weight; increased daily step counts by 24%; made fourfold increases in weekly METS expended in walking; decreased calories from fat by 2%, calories from sweets by 3%, and daily calories by 10%; and increased F&V intake by about 1.5 servings a day. These changes were consistent with those exhibited by users of a site-based version of GTH delivered with enhanced supports [18] and were preceded by improvements at 6 months in social support, self-efficacy, outcome expectations, and self-regulatory behaviors. 

A presumed strength of Internet interventions is that they can be highly flexible and personalized based on individual participant data [4]. In WB-GTH, users received program content at a time, pace, and setting determined by the user. Although the WB-GTH was just as effective with less-tailored, generic approaches to planning and feedback [32], the self-monitoring in both WB-GTH versions was quite detailed involving keeping and reporting daily behavioral logs. Bandura’s SCT [26,27] posits that adoption of and adherence to healthier eating and PA patterns is largely a matter of self-management; accordingly, SCT-based interventions should influence health behavior by influencing social support, self-efficacy, outcome expectations, and self-regulation of users. The current study provides an estimate of longitudinal effects of SCT variables within a complete theoretical model while simultaneously accounting for error in the measurement of variables. The SCT model provided good fit to nutrition, PA, and weight-management outcome data (RMSEA < .05). Consistent with other research, changes at 6 months in social support, self-efficacy, and self-regulation led to changes 10 months later in PA and, along with negative outcome expectations, in nutrition behavior [28]. In an advancement of previous investigations, weight loss was shown to be predicted by concomitant improvements in PA and nutrition; further, earlier changes in SCT variables contributed to weight loss largely through behavioral changes. In summary, WB-GTH users lost weight as they improved their diet and exercise habits resulting from enhanced psychosocial functioning.

The WB-GTH, like many other Internet programs, ultimately attracted a relatively affluent, predominantly female and white sample [29-31], many of whom declined to participate during the lengthy consent and enrollment process (~50% [29]). Research criteria designed to reduce risk associated with especially vigorous PA limited the external validity of the study by excluding elderly, unhealthy, and morbidly obese adults. Indeed, only about one-third of initially interested Web users qualified for the study [29]. Study exclusionary criteria were reported to have also had differential effects on Web users who were nonwhite [29]. Despite these limitations, the study’s inclusion criteria resulted in a sample of overweight or obese participants with step counts generally in the sedentary to inactive range (ie, <7500 steps/day), the vast majority of whom did not meet guidelines for intake of fat, fiber, and F&V [29].

Discussed in detail elsewhere [32], attrition in WB-GTH (59% baseline to 16 months) was associated with a number of tentative users who quickly withdrew from the study [21], with the extended length of the program, and with the extensive assessment component of the research project [32]. The WB-GTH shared early dropout and assessment procedures with the earlier site-based GTH trial that had a much lower attrition rate (22% attrition baseline to 16 months) [18]. Although the retained sample populations in the Web-based and site-based GTH programs were inactive to sedentary overall, dropouts from both versions [18,32] tended to be even less active, suggesting that generalizability of the study findings to the extremely sedentary may be limited. Seemingly differential attrition in the Web-based intervention suggests, however, that a shorter intervention may be more acceptable and face-to-face contact may contribute to higher retention. Internet program users, for example, may benefit from contact with program promoters such as health care providers, employers, clergy, or previous program users. Additionally, determining optimal program length for behavior-change maintenance may be key to retaining short-term and stable users [21].

Finally, although an earlier version of GTH was shown to be effective compared to an untreated control condition [18], there were no differences over time between participants using the two online versions of WB-GTH [32], thus limiting the study’s conclusions about the source of changes observed in weight, behavior, and psychosocial functioning. The effectiveness of the WB-GTH is arguably supported by outcomes that were sustained over time and by the limited effectiveness of similar programs in general [32]. Nevertheless, the pattern of psychosocial, behavioral, and anthropometric changes observed among WB-GTH users was consistent with the theoretical foundations of the GTH intervention, lending further credence to its being the source of those changes.

The longitudinal design of the study strengthened the structural equation analysis, but the ordering of the SCT variables was theoretical and not chronological [36]. The ordering, if correct, suggests that SCT interventions may be more effective if they increase social support and self-efficacy, and help participants set goals, plan, and monitor their nutrition and PA behaviors. Improving social support and self-efficacy may be an effective pathway for increasing the use of self-regulatory strategies. Further, improved social support, self-efficacy, and self-regulation may lead to improved PA levels and (along with outcome expectations) nutrition, which contribute to successful weight management among sedentary adults struggling with weight control. High initial levels of self-efficacy among Web-health users interested in online interventions may alter the function of SCT in these programs. The juxtaposition of high efficacy and expectations with low levels of healthy behavior is common. Bandura [26] suggests that self-efficacy for behavior change can be unrealistically high among individuals who lack experience in the desired, healthier behavior. While increased self-efficacy may enhance program outcomes, participants whose self-efficacy is tempered by online interventions may still benefit. After 6 months of using WB-GTH, for example, 51.5% (n = 140) of participants became less confident in their abilities to do the things necessary to be physically active on a regular basis (mean change -16.40, SD 16.61), 18% (n = 49) slightly increased their PA self-efficacy (mean change 4.39, SD 2.87), and 30% (n = 82) made larger improvements (mean change 23.89, SD 11.05). Participants who decreased self-efficacy during 6 months of WB-GTH started with significantly higher self-efficacy than those who gained the most confidence (mean 68.45, SD 18.69 vs mean 57.86, SD 19.27; *P* < .001). Participants at all three levels of change in self-efficacy, however, increased steps/day (mean 1167.05, SD 2854.75 vs mean 1501.61, SD 3851.76 vs mean 1806.88, SD 3253.27) and in METhours/week expended in walking-type activities (mean 7.26, SD 19.15 vs mean 8.73, SD 16.23 vs mean 10.12, SD 20.54). 

Although the current study demonstrates how SCT variables can contribute to the effectiveness of online interventions such as WB-GTH, researchers may need to go beyond traditional cognitive and motivational variables in order to explain a larger proportion of behavior change – perhaps, for example, assessing the affective and selective processes delineated in SCT that are associated with behavior change [26]. The WB-GTH, for example, tried to guide users to select social and physical environments that support their new behaviors (eg, finding a walking partner or eating at healthier restaurants) and WB-GTH’s guided mastery approach should have enhanced users’ abilities to anticipate and ameliorate aversive affective states associated with behavior change (eg, help users to like and feel good about their new healthier behaviors), but such effects were not measured in the trial.

The outcomes of WB-GTH suggest that purely Web-based interventions can operate in a manner consistent with underlying theory. Further, despite problems with attrition and a relatively homogeneous reach, Internet programs such as WB-GTH can be effective in guiding users to adopt healthier nutrition, PA, and weight-management habits, which rival changes produced by more intensive, face-to-face interventions.

## Abbreviations

F&V: fruits and vegetables
FIML: full information maximum likelihood
MANOVA: multivariate repeated measures analysis of variance
MET: metabolic equivalent
NOE: negative outcome expectations
PA: physical activity
RMSEA: root mean square error of approximation
SCT: social cognitive theory
SE: self-efficacy
SR: self-regulation
SS: social support
WB-GTH: Web-Based Guide to Health intervention
